# Normative values for the hypoparathyroidism patient questionnaire (HPQ28) in the German general population

**DOI:** 10.1186/s41687-025-00868-3

**Published:** 2025-04-03

**Authors:** Christian Trummer, Martina Blaschke, Deborah Quint, Maxi Schulz, Christoph Herrmann-Lingen, Matthias Büttner, Heide Siggelkow

**Affiliations:** 1 Clinic of Gastroenterology, Gastrointestinal Oncology and Endocrinology, University Medical Center Göttingen, Robert-Koch-Straße 40, 37075 Göttingen, Germany; 2 Division of Endocrinology and Diabetology, Department of Internal Medicine, Medical University of Graz, Auenbruggerplatz 15, Graz, 8036 Austria; 3 MVZ Endokrinologikum Göttingen, Von-Siebold-Straße 3, 37075 Göttingen, Germany; 4 Institute for Medical Statistics, University Medical Center Göttingen, Humboldtallee 32, 37073 Göttingen, Germany; 5 Clinic for Psychosomatic Medicine and Psychotherapy, University Medical Center Göttingen, Von-Siebold-Straße 5, 37075 Göttingen, Germany; 6 Institute of Medical Biostatistics, Epidemiology and Informatics (IMBEI), University Medical Center Mainz, Obere Zahlbacherstraße 69, 55131 Mainz, Germany; 7 University Cancer Centre, Langenbeckstraße 1, 55131 Mainz, Germany; 8 Department of Trauma, Orthopedics and Reconstructive Surgery, University Medical Center Göttingen, Robert-Koch-Straße 40, 37075 Götingen, Germany

**Keywords:** Hypoparathyroidism, Quality of life, Questionnaire, Symptom load, General population

## Abstract

**Background:**

Patients with hypoparathyroidism (HypoPT) suffer from several complaints and reduced quality of life (QoL), even if disease-specific biochemical parameters are within the target range. To be able to quantify symptoms in HypoPT patients, we recently developed a disease-specific questionnaire, the Hypoparathyroidism Patient Questionnaire with 28 items (HPQ28). The aim of this study was to find normative values for the HPQ28 in the German general population.

**Methods:**

We tasked an independent market and social research institute to obtain sociodemographic data and HPQ28 results from a representative sample of the German general population. The HPQ28 comprises five scales and three single items. The five scales indicate different areas of complaints: Pain and cramps (PaC) including five items, neurovegetative symptoms (NVS) including five items, loss of vitality (LoV) including six items, depression and anxiety (DaA) including five items, gastro-intestinal symptoms (GiS) including two items and two control items for depression. Three items were not attributable to any of the five scales: numbness and tingling in certain parts of the body (NT), troubled memory (TM), and racing heart (RH).

**Results:**

Mean age (± standard deviation) in the representative general population sample (*n* = 2506) was 49.5 ± 17.8 years, 51% were female. All scales and single items were affected by gender with women presenting significantly more complaints on every scale and single item in comparison to men (*p* < 0.01, Mann-Whitney U test). In addition, all scales and single items, except for GiS, were affected by age in males and females (*p* < 0.001, Spearman’s correlation). Regression analyses proved a linear trend in the different scores regarding age and gender (*p* < 0.05 except for age on the GiS scale).

**Conclusions:**

We present data from the first application of the HPQ28 in a representative sample of the German general population. Almost all scales and single item of the HPQ28 were dependent on age and gender, with older individuals and females presenting a higher burden of complaints.

**Trial registration:**

DRKS, DRKS00027581. Registered 17th of January 2022, https//drks.de/search/de/trial/DRKS00027581.

**Supplementary Information:**

The online version contains supplementary material available at 10.1186/s41687-025-00868-3.

## Background

Hypoparathyroidism (HypoPT) is an endocrine disorder defined as inadequately low or undetectable parathyroid hormone (PTH) secretion from the parathyroid glands accompanied by low concentrations of corrected total calcium or ionized calcium in serum [1]. Most cases (approximately 75%) occur postoperatively after neck surgery, while nonsurgical etiologies include autoimmune and genetic causes (e.g., DiGeorge syndrome) [2]. Conventional therapy of HypoPT consists of calcium supplements and active vitamin D analogues with the aim of achieving serum calcium levels in the low normal range or slightly below, while also normalizing serum phosphorus and magnesium concentrations as well as urine calcium excretion [3]. Patients for whom conventional therapy proves insufficient may be offered replacement therapy with recombinant human PTH (1–84), novel substances (e.g. TransCon PTH, palopegteriparatide) are currently under investigation [3, 4].

In addition to typical symptoms of hypocalcemia (e.g. paresthesia, muscle cramps), patients also suffer from neurocognitive and psychologic impairment, even when target serum calcium levels are achieved [5, 6]. Especially in patients undergoing standard treatment, fluctuation in serum calcium concentrations, impaired renal function, and reduced quality of life (QoL) are commonly reported [3]. Thus, HypoPT represents a complex endocrine condition that cannot be assumed to be treated adequately solely by controlling serum calcium concentrations [7]. To assess the impact of HypoPT on patients’ individual clinical symptoms as well as on QoL, a variety of tools were studied and implemented. These included the Short Form 36 Health Survey (SF-36), the WHO-5 Well-Being Index Survey (WHO-5), the Hospital Anxiety and Depression Scale (HADS), the revised Symptom Checklist 90 (SCL-90-R), and the short form of the Giessen Complaint List (GBB-24) [6, 8–11]. None of these tools or questionnaires, however, was designed specifically with typical symptoms of HypoPT in mind, while a questionnaire conceptualized particularly for use in HypoPT might have improved performance in detecting disease-specific symptoms as well as changes over time [12]. In addition to instruments developed by other groups, such as the Hypoparathyroidism Patient Experience Scale Symptom (HPES-Symptom) [13–15], Wilde et al. [16] proposed the use of the Hypoparathyroidism Patient Questionnaire with 28 items (HPQ28) as a HypoPT-specific tool to evaluate subjective symptoms and complaints. In patients with postsurgical HypoPT, the HPQ28 was able to identify and quantify typical symptoms successfully when compared to patients after thyroid surgery that did not develop HypoPT as well as patients with primary hyperparathyroidism [17]. In HypoPT, a correlation between complaints measured by the HPQ28 and biochemical parameters could be demonstrated [17]. Data on the effects of different treatment modalities on subjective symptoms according to the HPQ28 were also published previously, suggesting that the reduced QoL in HypoPT may be caused in part by the use of conventional treatment with calcium and active vitamin D supplements [18].

While the benefit of the HPQ28 in patients with HypoPT is well documented, data from the general population are currently lacking. Normative values are of particular interest, given the fact that age and gender influence a number of other questionnaires [19]. Thus, the aim of this study was to apply the HPQ28 in a large representative sample of the German general population to develop normative values for comparison with patient groups, as well as have the option of characterizing the symptom burden of single patients.

## Methods

### Study design and study participants

This study was designed as cross-sectional on a representative sample of the German general population. Thus, we commissioned USUMA GmbH (Unabhängiger Service für Umfragen, Methoden und Analysen, Berlin, Germany) as an independent market and social research institute to conduct 2500 face-to-face interviews in persons aged 16 years or older within the German general population. To achieve utmost conformity with the German general population, we did not screen for any prevalent diseases including HypoPT. The survey took place in October and November 2021. In brief, by applying the Kish selection grid, participants per household were chosen randomly and informed about the study verbally as well as in writing. Initially, 5934 households were chosen for the survey and 5901 of the obtained addresses could be verified. Among these, 25% refused to participate, while 21% could not be contacted. In summary, 2526 face-to-face interviews could be completed, although 17 interviews failed to meet the necessary criteria for adequate analysis. This resulted in 2509 interviews that could be used for study purposes with completed HPQ28 questionnaires. The main part of the survey comprised a questionnaire in which participants were asked to provide sociodemographic data and the HPQ28. All study participants filled out the questionnaire independently and without any default input by the interviewer, who assisted if any questions arose. This resembles the use of the HPQ28 in HypoPT patients in clinical practice, as patients may receive assistance from medical personnel if needed. All questionnaires were in German language and all interviews were also conducted in German. To assure the reliability of the interviews, postcards were sent randomly to 45% of the participants. A total of 58.9% of the postcards were returned, all confirming proper interview conduction. To achieve two gender groups, three persons who identified as neither male nor female were excluded, resulting in data from 2506 individuals that were used for statistical analysis. All data were handled in accordance with the European General Data Protection Regulation. The institutional review board of University Medical Center Göttingen approved the study (approval number: 25/10/15).

### Questionnaires

Questionnaires handed out to study participants were divided in two parts. During the first part, standardized individual and household related sociodemographic data were obtained. The second part consisted of the HPQ28 questionnaire designed to evaluate the disease-specific symptoms and complaints in patients with HypoPT [16].

Originally 40 questions from generic questionnaires administered in patients with hypoPT and significantly different from normative controls were rephrased when needed, and then structured as the preliminary disease characteristic HPQ 40. After being reviewed for structure and completeness by an endocrinologist as well as a panel of psychologic experts, and after testing in healthy adults, it was prospectively investigated in three patient groups, one of them hypoPT patients. If items on the questionnaire would be specific for HypoPT patients, the answers to the items should be different from the other two groups.

In the next step we wanted to analyze if there are domains representing several items. Due to the very different symptoms described by the patients and, hypothetically, missing symptoms in two control groups, we hypothesized only little correlation between the different items. We applied PCA (principle component analysis) for the initial design of the HPQ28 questionnaire, as a method to reduce the set of items. PCA is a frequently applied method and easy to perform. The assumed little correlation between the items justifies using PCA which relies on the use of the correlation matrix. A high correlation is not expected in HPQ28 questionnaire.

An orthogonal Varimax rotation was chosen with the idea, that items belong to groups (symptom domains, further termed as “scales“) which are ideally in theory uncorrelated and therefore independent explaining the variance of the symptoms. Due to the fact that in some scales the item number was more than five items, we wanted to reduce redundant items. By calculating the factor loading of the different items we were able to reduce the number of items on the questionnaire accordingly which resulted in the end in 28 items explaining the name “HPQ28”.

We do not know if using another factor analysis method would have given other results. Meanwhile the specificity of our analysis was confirmed in other patient groups by good correlation to other generic tools [20], confirmatory analysis of the French version [21] and correlation with changes in brain function with the Danish version of the HPQ28 [22].

The HPQ28 now comprises five scales and three single items. Each scale focuses on a different area of complaints: Pain and cramps (PaC) including five items (items 3, 6, 12, 14, and 20), neurovegetative symptoms (NVS) including five items (items 4, 11, 16, 17, and 19), loss of vitality (LoV) including six items (items 23–28), depression and anxiety (DaA) including five items (items 7, 9, 13, 15, and 18), gastro-intestinal symptoms (GiS) including two items (items 8 and 10). Two additional items (21–22) were taken from the Patient Health Questionnaire (PHQ-2) as an established screening tool for depression [23]. Three single items were not attributable to any of the five scales (items 1, 2, and 5), i.e. numbness and tingling in certain parts of the body (NT), troubled memory (TM), and racing heart (RH). The scoring system works on a four-step scale from 0 (“not at all”) to 3 (“severely”) as indication of symptom intensity. Results of the application of the HPQ28 questionnaire in HypoPT patients were published previously [17].

### Statistical analysis

All statistical analyses were performed with IBM-SPPS software version 29 (IBM Corp., Armonk, NY, USA). Answers to items of the HPQ28 were coded as 0 = not at all, 1 = slightly, 2 = moderately, or 3 = severely [16]. Characteristics of the sample of the German general populations were compared between female and male participants using Student`s t-test for continuous variables (age) or the chi-squared test for categorical variables. To test for differences between female and male participants in subcategories, we performed post-hoc pairwise comparison using the Bonferroni correction method. Participants were divided into seven age groups per gender (< 25, 25–34, 35–44, 45–54, 55–64, 65–74, and ≥ 75 years, respectively). Spearman`s test was used to determine any possible correlation between age and the results of the scales and single items of the HPQ28 in both genders. To account for multiplicity in the analysis, we applied Bonferroni correction to the p-values obtained from the distinct regression analyses for each scale (F-statistic) and compared the adjusted p-values to a significance level of 0.05, thereby ensuring a fixed global family-wise error rate. If regression models remained significant after adjustment, we included them in the interpretation and discussion. Further exploratory tests are not adjusted for multiplicity.

## Results

The mean age (± standard deviation, SD) in our sample of the German general population (*n* = 2506) was 49.5 ± 17.8 years, and 51% were female. Participants were divided into seven age groups with equal gender distribution (Table 1). Regarding the socio-ethnic background, 96% of the sample size were of German nationality, 44% were living in a partnership, 35% achieved an intermediate school-leaving certificate (*“Mittlere Reife”*) as their highest form of education, 46% were full-time employees, and the largest religious group was of protestant Christian faith (36%). Table 1 gives an overview of the general population sample size characterization. Supplemental Figure S1 portrays the overall distribution of obtained HPQ28 scale and single items points across the entire study population group.

**Table 1:** Characteristics of the German general population sample

	All (*n* = 2506)	Female (*n* = 1276)	Male (*n* = 1230)	*p*-value
Age (years)	49.5 ± 17.8	49.2 ± 17.8	49.9 ± 17.8	0.33
*Age groups* <25 years 25–34 years 35–44 years 45–54 years 55–64 years 65–74 years ≥75 years	228 (9%) 382 (15%) 391 (16%) 464 (19%) 480 (19%) 341 (14%) 220 (9%)	120 (9%) 201 (16%) 193 (15%) 246 (19%) 243 (19%) 161 (13%) 112 (9%)	108 (9%) 181 (15%) 198 (16%) 218 (18%) 237 (19%) 180 (15%) 108 (9%)	0.72
*Nationality* German Other	2416 (96%) 90 (4%)	1237 (97%) 39 (3%)	1179 (96%) 51 (4%)	0.59
*Civil status* In partnership Single Divorced Widowed Married, living separated	1106 (44%) 763 (31%) 367 (15%) 208 (8%) 58 (2%)	535 (42%)* 347 (27%)* 208 (16%)* 152 (12%)* 32 (3%)	571 (46%)* 416 (34%)* 159 (13%)* 56 (5%)* 26 (2%)	< 0.001
*Highest education degree* No secondary education Basic school-leaving certificate (“Hauptschulabschluss”) Intermediate school-leaving certificate („Mittlere Reife“) Graduation from polytechnic Secondary school (East Germany) Graduation from vocational training School Highest school-leaving certificate (“Abitur) Higher education (university, “Fachhochschule”) Currently in school/higher education	52 (2%) 317 (25%) 871 (35%) 237 (9%) 117 (5%) 334 (13%) 231 (9%) 46 (2%)	32 (3%) 306 (24%) 474 (27%)* 100 (8%)* 52 (4%) 170 (13%) 117 (9%) 24 (2%)	20 (2%) 311 (25%) 397 (32%)* 137 (11%)* 65 (5%) 164 (13%) 114 (9%) 22 (2%)	0.028
*Employment* Full-time (> 35 h/week) Part-time (15–34 h/week) Retirement In education/training Unemployed Other	1148 (46%) 310 (12%) 622 (25%) 156 (6%) 120 (5%) 145 (6%)	455 (36%)* 264 (24%)* 302 (24%) 77 (7%) 49 (4%)* 125 (10%)*	693 (56%)* 46 (5%)* 320 (26%) 79 (6%) 71 (6%)* 20 (2%)*	< 0.001
*Type of employment* Independent contractor Employee Public official Worker/laborer No employment yet	140 (6%) 1471 (59%) 133 (5%) 561 (23%) 149 (6%)	41 (3%)* 959 (75%)* 55 (4%)* 110 (9%)* 86 (7%)	99 (7%)* 512 (42%)* 78 (7%)* 451 (37%)* 63 (5%)	< 0.001
*Confession* No confession Protestant Catholic Islam Other	752 (30%) 908 (36%) 689 (28%) 73 (3%) 56 (3%)	317 (25%)* 511 (40%)* 367 (29%) 41 (3%) 27 (2%)	435 (35%)* 397 (32%)* 322 (26%) 32 (3%) 29 (2%)	< 0.001

Data are illustrated as means ± standard deviation or absolute number of individuals (n) with percentages, as appropriate. Comparisons between female and male participants were performed using Student’s t-test or the chi-squared test, as appropriate. * denotes a significant difference between female and male participants in post-hoc pairwise comparison after Bonferroni correction

**Fig. 1:**
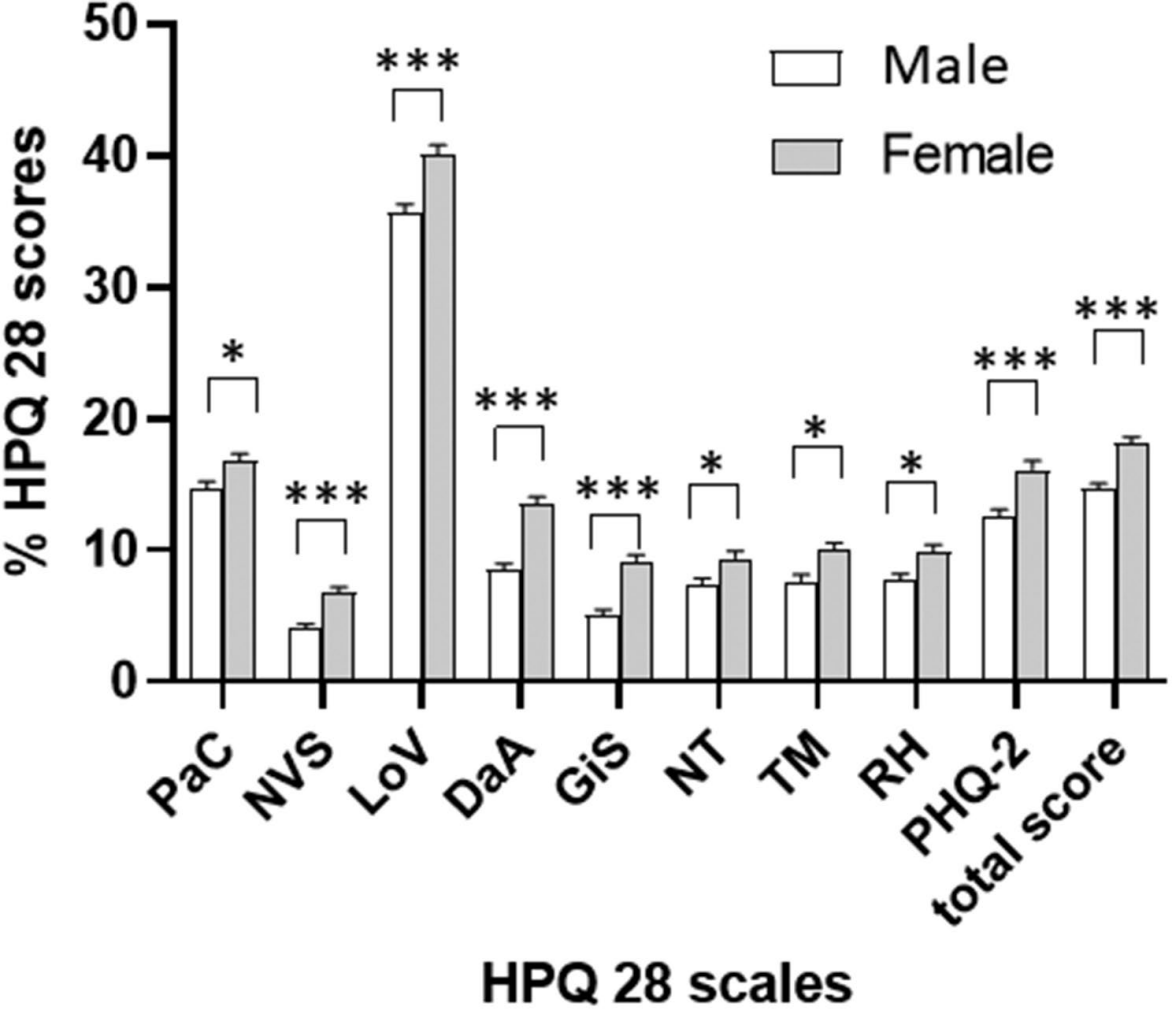
HPQ28 scores (%) classified according to the five scales (PaC, NVS, LoV, DaA, GiS), the three single items (NT, TM, RH) and the PHQ-2 depression scale as well as the total HPQ28 score in female and male participants. Differences between genders were calculated using Student’s t-test, *p < 0.05, ***p < 0.001 and are multiplicity adjusted for 9 scales as well as for gender (Fig. 1) and age (Fig. 2). DaA = depression and anxiety, GiS = gastrointestinal symptoms, HPQ28 = 28 item Hypoparathyroidism Patient Questionnaire, LoV = loss of vitality, NT = numbness or tingling, NVS = neurovegetative symptoms, PaC = pain and cramps, PHQ-2 = 2 item Patient Health Questionnaire, RH = racing heart, TM = troubled memo

**Fig. 2:**
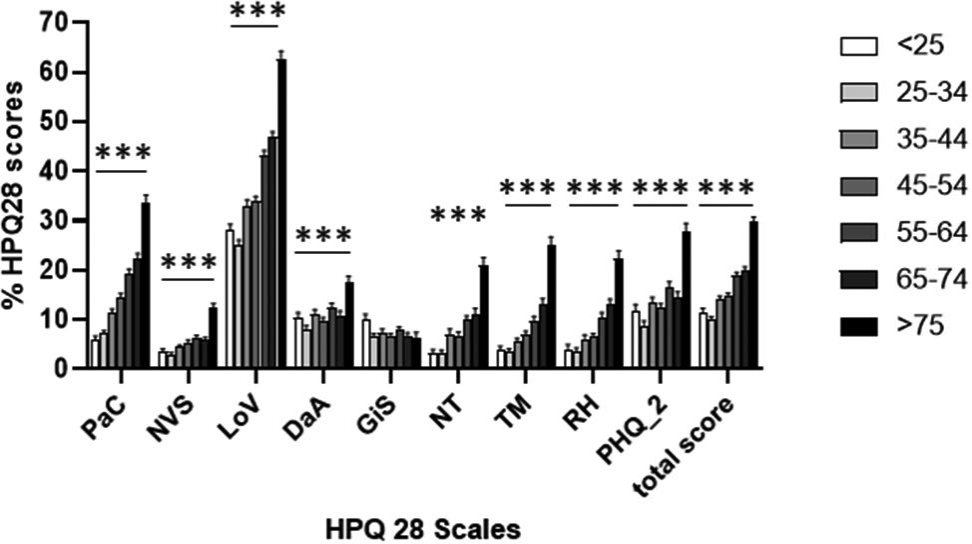
HPQ28 scores (%) classified according to the five scales (PaC, NVS, LoV, DaA, GiS), the three single items (NT, TM, RH) and the PHQ-2 depression scale as well as the total HPQ28 score in seven different age groups. Differences between age groups were calculated using One-Way-ANOVA, ***p < 0.001 and are multiplicity adjusted for 9 scales as well as for gender (Fig. 1) and age (Fig. 2). DaA = depression and anxiety, GiS = gastrointestinal symptoms, HPQ28 = 28 item Hypoparathyroidism Patient Questionnaire, LoV = loss of vitality, NT = numbness or tingling, NVS = neurovegetative symptoms, PaC = pain and cramps, PHQ-2 = 2 item Patient Health Questionnaire, RH = racing heart, TM = troubled memory

All scales and items of the HPQ28 were significantly influenced by gender (Table 2; Fig. 1): in our sample of the German general population, women reported significantly more complaints on every scale and single item of the HPQ28 when compared to men (*p* < 0.01 for all). Furthermore, complaints measured by the scales PaC, NVS, LoV, DaA, the single items (NT, TM, RH) as well as the PHQ-2 significantly increased with age in both females and males (*p* < 0.001, Table 3; Fig. 2). The only scale not significantly affected by age was the GiS scale (Table 3). All figures portray percentages instead of HPQ28 points to improve legibility (with 100% representing the maximum of points obtainable, i.e. the highest possible symptom load).

**Table 2:** HPQ28 means and standard deviation in the German general population sample according to age and gender

HPQ28 scales or items^1^	Age group (years)								
	< 25	25–34	35–44	45–54	55–64	65–74	≥ 75	All Participants	*p*-value male vs. female
*PaC (15)*									
Male	0.6 ± 1.2	0.9 ± 1.6	1.5 ± 2.3	2.1 ± 2.3	2.5 ± 2.5	3.2 ± 2.7	5.1 ± 3.1	2.2 ± 2.6	0.007
Female	1.0 ± 2.2	1.3 ± 2.2	1.8 ± 2.4	2.3 ± 2.7	3.2 ± 3.0	3.5 ± 2.7	5.1 ± 3.0	2.5 ± 2.9	
*NVS (15)*									
Male	0.3 ± 0.8	0.2 ± 0.7	0.5 ± 1.3	0.6 ± 1.5	0.7 ± 1.5	0.6 ± 1.3	1.5 ± 2.0	0.6 ± 1.4	< 0.001
Female	0.7 ± 1.7	0.6 ± 1.5	0.8 ± 1.5	1.0 ± 1.7	1.2 ± 1.7	1.0 ± 19	2.1 ± 2.2	1.0 ± 1.8	
*LoV (18)*									
Male	4.3 ± 2.9	3.8 ± 3.6	5.6 ± 4.7	5.8 ± 4.0	7.2 ± 4.3	8.1 ± 3.7	11.3 ± 3.6	6.5 ± 4.5	< 0.001
Female	5.9 ± 4.0	5.2 ± 3.9	6.3 ± 4.3	6.4 ± 4.3	8.3 ± 4.3	8.9 ± 4.1	11.7 ± 4.0	7.3 ± 4.5	
*DaA (15)*									
Male	0.9 ± 2.0	0.8 ± 1.8	1.4 ± 2.6	1.1 ± 1.9	1.5 ± 2.4	1.2 ± 1.9	2.1 ± 2.6	1.3 ± 2.2	< 0.001
Female	2.1 ± 2.8	1.5 ± 2.8	1.9 ± 2.7	1.7 ± 2.7	2.2 ± 2.9	2.0 ± 2.8	3.1 ± 3.2	2.0 ± 2.9	
*GiS (6)*									
Male	0.3 ± 0.7	0.2 ± 0.5	0.3 ± 0.8	0.3 ± 0.8	0.4 ± 0.9	0.3 ± 0.7	0.2 ± 0.6	0.3 ± 0.8	< 0.001
Female	0.8 ± 1.3	0.6 ± 1.0	0.6 ± 1.0	0.4 ± 0.9	0.5 ± 1.0	0.5 ± 1.0	0.5 ± 1.0	0.5 ± 1.0	
*NT (3)*									
Male	0.02 ± 0.3	0.03 ± 0.2	0.2 ± 0.6	0.2 ± 0.5	0.2 ± 0.5	0.3 ± 0.6	0.6 ± 0.7	0.2 ± 0.5	0.002
Female	0.2 ± 0.4	0.2 ± 0.5	0.2 ± 0.6	0.2 ± 0.6	0.3 ± 0.6	0.3 ± 0.6	0.7 ± 0.7	0.3 ± 0.6	
*TM (3)*									
Male	0.05 ± 0.3	0.05 ± 0.3	0.1 ± 0.5	0.2 ± 0.4	0.2 ± 0.5	0.4 ± 0.5	0.8 ± 0.8	0.2 ± 0.5	< 0.001
Female	0.2 ± 0.5	0.2 ± 0.5	0.2 ± 0.5	0.2 ± 0.5	0.3 ± 0.6	0.4 ± 0.6	0.7 ± 0.8	0.3 ± 0.6	
*RH (3)*									
Male	0.05 ± 0.3	0.08 ± 0.3	0.1 ± 0.5	0.2 ± 0.4	0.3 ± 0.6	0.4 ± 0.6	0.6 ± 0.7	0.2 ± 0.5	< 0.001
Female	0.2 ± 0.5	0.1 ± 0.4	0.2 ± 0.5	0.2 ± 0.5	0.3 ± 0.6	0.4 ± 0.6	0.7 ± 0.7	0.3 ± 0.6	
*PHQ-2 (6)*									
Male	0.4 ± 0.8	0.4 ± 0.8	0.8 ± 1.4	0.7 ± 1.1	0.9 ± 1.3	0.8 ± 1.1	1.5 ± 1.5	0.8 ± 1.2	< 0.001
Female	1.0 ± 1.4	0.7 ± 1.3	0.8 ± 1.3	0.8 ± 1.3	1.1 ± 1.4	1.0 ± 1.3	1.8 ± 1.7	1.0 ± 1.4	
*Total (84)*									
Male	7.1 ± 7.0	6.3 ± 7.4	10.7 ± 11.4	11.1 ± 9.7	13.9 ± 11.4	15.3 ± 9.2	23.7 ± 11.8	12.3 ± 11.0	< 0.001
Female	12.2 ± 11.6	10.3 ± 11.1	12.9 ± 11.5	13.3 ± 11.9	17.5 ± 12.7	18.1 ± 11.7	26.4 ± 13.1	15.2 ± 12.7	

^1^Bracketed numbers represent the maximum of points obtainable in either scale or item. Data are illustrated as mean ± standard deviation even though data are non-normally distributed to improve legibility. p-values are given for comparisons between all males and females and were calculated using the Mann-Whitney U test. DaA = depression and anxiety, GiS = gastrointestinal symptoms, HPQ28 = 28 item Hypoparathyroidism Patient Questionnaire, LoV = loss of vitality, NT = numbness or tingling, NVS = neurovegetative symptoms, PaC = pain and cramps, PHQ-2 = 2 item Patient Health Questionnaire, RH = racing heart, TM = troubled memory

**Table 3:** Spearman correlation between scale or single item results of the HPQ28 and age in both female and male participants

Scale/single item	Female (*n* = 1276)	Male (*n* = 1230)	All (*n* = 2506)
	ρ	ρ	ρ
PaC	0.45	0.48	0.46
NVS	0.22	0.23	0.22
LoV	0.38	0.45	0.41
DaA	0.13	0.15	0.14
GiS	ns	ns	ns
NT	0.24	0.31	0.27
TM	0.27	0.35	0.31
RH	0.26	0.31	0.28
PHQ-2	0.17	0.22	0.19

All correlations (except GiS) were highly significant (p-values < 0.0001). Due to high number of samples, p-values are not informative and not given

DaA = depression and anxiety, GiS = gastrointestinal symptoms, HPQ28 = 28 item Hypoparathyroidism Patient Questionnaire, LoV = loss of vitality, ns = non-significant, NT = numbness or tingling, NVS = neurovegetative symptoms, PaC = pain and cramps, PHQ-2 = 2 item Patient Health Questionnaire, RH = racing heart, TM = troubled memory

Linear regression analyses with age and gender as independent variables proved a significant relation between age and gender and the different scores (*p* < 0.05), except for age on the GiS scale, Table 4). The adjusted R2 values for scales PaC and LoV resulted in low values (Table 4), indicating that the data are not well explained by the regression model. One reason for this could be that other important variables explaining the outcomes for the remaining scales and items are missing in the analysis. However, these adjusted R2 values are comparable to other studies in the field. The low regression coefficients may be misleading. The regression coefficients represent the mean corresponding score change for gender (= 1 female) and one age group of seven age groups and depends on the maximum score of each scale or item. Therefore, as scores for the 5 scales (PaC, NVS, DaA (all maximum score 15 points), LoV (18 points), GiS (6 points) and the 3 single items (maximum score 3 points) as well as scale PHQ-2 (6 points) vary, the regression coefficients must be valued respectively. In addition, we now provide the maximum score for every scale in Table 4. After applying Bonferroni correction for multiplicity, all regression analyses proved significant at a global significance level of 0.05.

**Table 4:** Results of linear regression analyses with age and gender as independent variables

	PaC max 15 points			
	B	Beta	Significance	Adjusted R^2^
Lin. Model				0.178
Constant *k*	-1.04			
Gender Female	0.369	0.067	< 0.001	
Age	0.065	0.418	< 0.001	
	NVS max 15 points			
Lin. Model				0.054
Constant *k*	-0.28			
Gender Female	0.422	0.131	< 0.001	
Age	0.018	0.200	< 0.001	
	LoV max 18 points			
Lin. Model				0.170
Constant *k*	1.31			
Gender Female	0.904	0.100	< 0.001	
Age	0.103	0.403	< 0.001	
	DaA max 15 points			
Lin. Model				0.032
Constant *k*	0.49			
Gender Female	0.761	0.147	< 0.001	
Age	0.016	0.107	< 0.001	
	GiS max 6 points			
Lin. Model				0.018
Constant *k*	0.364			
Gender Female	0.246	0.136	< 0.001	
Age	-0.001	-0.025	0.21	
	NT max 3 points			
Lin. Model				0.059
Constant *k*	-0.160			
Gender Female	0.069	0.060	0.002	
Age	0.008	0.239	< 0.001	
	TM max 3 points			
Lin. Model				0.086
Constant *k*	-0.226			
Gender Female	0.079	0.070	< 0.001	
Age	0.009	0.287	< 0.001	
	RH max 3 points			
Lin. Model				0.077
Constant *k*	-0.187			
Gender Female	0.075	0.068	< 0.001	
Age	0.008	0.271	< 0.001	
	PHQ-2 max 6 points			
Lin. Model				0.040
Constant *k*	0.089			
Gender Female	0.227	0.088	< 0.001	
Age	0.013	0.183	< 0.001	

B: Regression coefficient; Beta: standardized coefficient; DaA = depression and anxiety, GiS = gastrointestinal symptoms, HPQ28 = 28 item Hypoparathyroidism Patient Questionnaire, LoV = loss of vitality, NT = numbness or tingling, NVS = neurovegetative symptoms, PaC = pain and cramps, PHQ-2 = 2 item Patient Health Questionnaire, RH = racing heart, TM = troubled memory

A possible application for calculating normative values regarding age and gender using data from the regression analysis for research purposes is available in the supplementary file.

Supplemental Table 1 shows percent ranks of the HPQ28 scales and single items by gender and age.

## Discussion

In this cross-sectional study, we tested a representative sample of the German general population with the HPQ28, a questionnaire specifically designed to evaluate subjective symptoms and complaints in patients presenting HypoPT. All scales and items of the questionnaire were influenced by gender with females reporting significantly more symptoms. The scores of all the scales and items except for GiS also increased with age. Finally, linear regression analysis proved that both age and gender have a significant influence on the different scores, the only exception being the GiS scale with respect to age.

The generation of normative data is essential to allow in-depth comparison between diverse samples of subjects and thorough outcome assessments [24]. The obtained data may prove helpful if other control groups are difficult to form due to, as in low disease prevalence for example, which is the case in HypoPT [25]. Furthermore, comparisons with normative data have been shown to be a motivational factor for patients to change their behavior and seek professional help [26]. This could also be of relevance to HypoPT patients, since symptoms often persist even when target values for biochemical parameters have been achieved [7]. As patients with HypoPT often report that their doctors frequently underestimate or dismiss the burden of their symptoms, the use of disease-specific scores like the HPQ28 in combination with normative values could also improve the communication between patients and healthcare professionals [3].

The validity of our study sample as a representative depiction of the German general population is underscored by comparisons with data published by the German Federal Statistical Office *(Statistisches Bundesamt*,* Destatis*). In our sample, we observed a similar proportion of women (51% vs. 51%) [27], a comparable proportion of persons living in partnerships (44% vs. 42%) [28], however a difference in unemployment rates (4.8% vs. 5.9%) [29] when compared to the data provided by the federal agency. Of note, the proportion of study subjects with any other nationality except German was relatively low (4%) when compared to the official data (12.8%) [27]. This fits well with experience in many Western countries that persons with a migrant background tend to be underrepresented in health surveys and generally respond less often to population-based surveys [30]. When female study participants were compared to male subjects, women were more likely to be widowed, in part-time employment, or in other forms of occupation (e.g., maternity leave), while more women were in a classical employee-employer relationship.

Scores of all the HPQ28 scales and items except for GiS increased with age, indicating a higher burden of complaints in older individuals. These findings confirm observations previously made in the general population. For example, in concordance with the results of the PaC scale in the HPQ28 in this study, numerous other research groups have reported an increase in pain in older individuals [31, 32]. Likewise, an increase in depression and anxiety (measured by the HPQ28 DaA scale) with age has been postulated by others using different measurement tools, e.g. the Brief Symptom Inventory with 18 items (BFI-18) [33]. Reflecting the increased burden with age on the LoV scale in this study, other questionnaires commonly used to assess health-related quality of life (e.g., the 36-Item Short Form Health Survey, SF-36) are also known to reveal a decline in physical well-being in the elderly [34]. Measures of fatigue like the Multidimensional Fatigue Inventory also decline with older age [35]. The missing impact of age on the GiS scale may be explained by the higher prevalence of certain gastrointestinal disorders, e.g. functional disorders, in younger patients [36]. Furthermore, there is evidence of an age-related decrease in the perception of abdominal pain [37].

We found higher HPQ28 scores for every scale and single item in women when compared to male participants. This also confirms existing data revealing that women have higher prevalence rates of pain [31] as well as higher rates of depression and anxiety [38, 39]. When filling out the Giessen Subjective Complaints List (GBB-8), women were also shown to present more somatic symptoms [40]. Comparable to the HPQ28, this questionnaire contains scales evaluating abdominal discomfort, palpitations, exhaustibility, neck/shoulder pain and back/sacroiliac pain [40]. Furthermore, several abdominal disorders such as irritable bowel syndrome are more common in women than in men, which may at least in part explain the differences we found between genders in the HPQ28 GiS scale [41].

Due to the large size of our sample of the German general population with over 2500 study subjects, one must exercise caution when interpreting the observed significant clinical differences between groups. For example, group differences between female and male participants in HPQ28 single items (NT, TM, RH) were comparatively small, however, yielded highly significant p-values in view of the large sample size. Thus, we cannot rule out with certainty that the clinical impact of these findings is negligible. This should be taken into consideration when our data is interpreted for clinical purposes.

Our study has several strengths and limitations that should be observed when interpreting the results. As a cross-sectional study, the results only represent the condition of study participants at a set point in time (in our case, October-November 2021). This implies that the results may have been different if the study was conducted at a different point in time [42]. While it is preferable to recruit participants in a random manner over, e.g., advertising, this may lead to the problem of low response rates and small group sizes [42]. In this study, the response rate was decent with 2526 completed interviews out of 5901 contacted households in total (43%). However, we clearly cannot rule out that the non-responders had particularly high or low HPQ28 scores, thus leading to a possible underestimation or overestimation of the true population normative (e.g. caused by higher rates of non-responders in certain population groups, as discussed previously). Since the interviews were conducted during the COVID pandemic in Germany (October-November 2021), this may influence on HPQ28 results adversely. Furthermore, as a study conducted in the German general population, our results may not carry over to general populations in other countries.

Strengths of our study include its large sample size that allows us to draw conclusions on normative values within the German general population as a whole. The representativeness of our data for the German general population is underlined by several characteristics that are found in our data as well as in data from the general population. Additionally, this is the first study to apply the HPQ28 in a sample population of the general population.

## Conclusions

We present data from the first application of the HPQ28 in a sample representative of the German general population. Almost all scales and single items of the HPQ28 proved to be dependent on age and gender, with older individuals and females presenting a higher burden of complaints.

## Electronic supplementary material

Below is the link to the electronic supplementary material.

Supplementary Material 1

## Data Availability

The datasets used and/or analyzed during the current study are available from the corresponding author on reasonable request.

## Abbreviations

BFI 18: Brief symptom inventory with 18 items
DaA: Depression and anxiety
GBB: Giessen complaint list
GiS: Gastrointestinal symptoms
HADS: Hospital anxiety and depression scale
HPES: Symptom hypoparathyroidism patient experience scale symptom
HPQ28: Hypoparathyroidism patient questionnaire with 28 items
HypoPT: Hypoparathyroidism
LoV: Loss of vitality
NT: Numbness or tingling
NVS: Neurovegetative symptoms
PaC: Pain and cramps
PHQ-2: Patient health questionnaire
PTH: Parathyroid hormone
QoL: Quality of life
RH: Racing heart
SCL-90-R: Revised symptom checklist 90
SD: Standard deviation
SF-36: Short form 36 health survey
TM: Troubled memory
WHO-5: WHO-5 Well-being index survey
