# Supplementary material for: Normative values for the hypoparathyroidism patient questionnaire (HPQ28) in the German general population
Source: J Patient Rep Outcomes. 2025 Apr 3;9:38. doi: 10.1186/s41687-025-00868-3 (PMC11968587; doi:10.1186/s41687-025-00868-3)
Supplement: Supplementary file 1 — Supplementary Material 1 [file 41687_2025_868_MOESM1_ESM.docx]

Supplemental File

**Normative Values for the Hypoparathyroidism Patient Questionnaire (HPQ28) in the German General Population**

Distribution of obtained scale and single items points across all study participants (Fig S1):


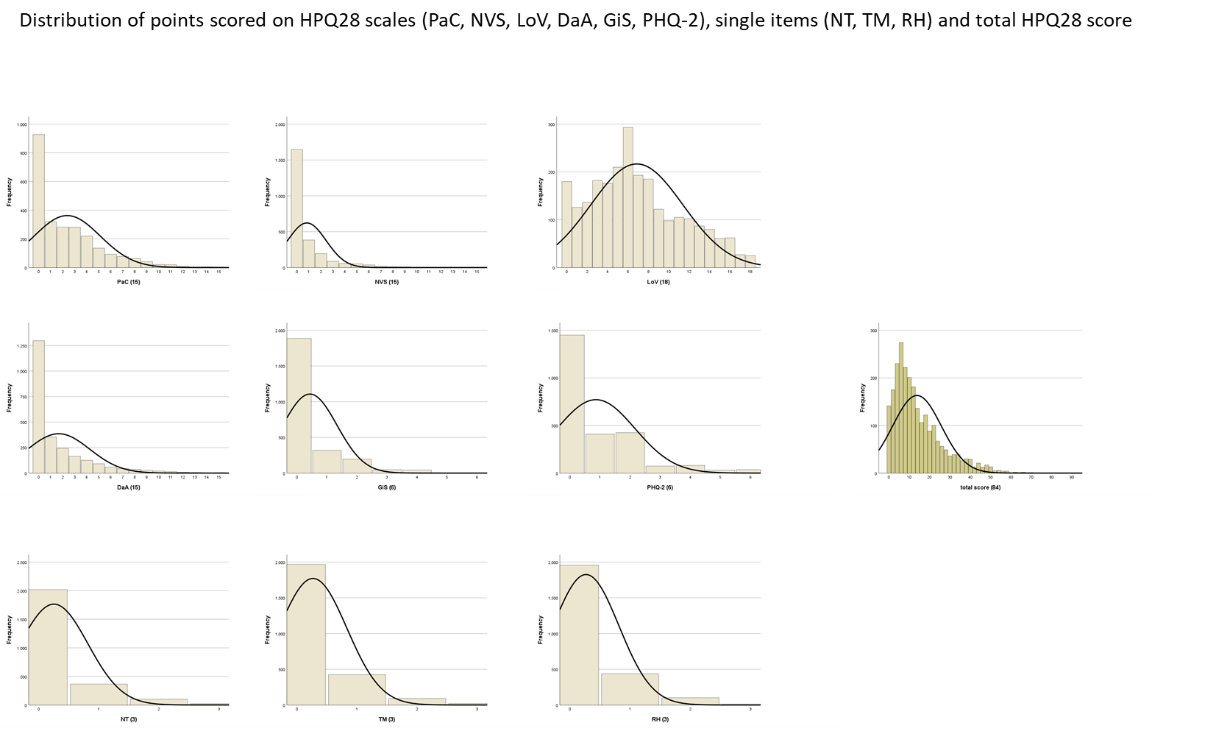


Fig S1: Histogram and Density Plot for each scale and single item

Linear Regression:

Generated variables by linear regression analysis allow the calculation of comparative values adjusted to each patient population or single patient. The advantage of normative values using a regression formula lies in the integration of age and gender effects. Using this calculation method, comparisons between different patient groups with age and gender differences among groups will be possible, as also demonstrated for other questionnaires (19). To interpret the results, the difference between the patients’ mean scores and the calculated score may serve as an indicator of the burden of HypoPT symptoms

Using the parameters depicted in Table 4 of the main manuscript, normative values for each HPQ28 scale and item can be calculated using the following formula:

*Normative HPQ28 scale/item = k* + *(proportion of male-female)* x *a* + *age* x *b,*

where *k* represents a constant parameter for the intersection of the imaginary line with the y-axis, *a* represents the coefficient of the gender effect, and *b* represents the coefficient of the effect of age. For example, if a given patient sample is characterized by a mean age of 65 years with a female proportion of 50%, the normative value for the PaC scale for comparison with the patient population is calculated as:

***-****1.04 + 0.50 x (0.369) + 65 x (0.065) = 3.4*

*(constant k + proportion of male-female x coefficient a + mean age x coefficient b)*

Thus, in the German general population with a mean age of 65 years and a female proportion of 50%, a normative value of 3.4 on the PaC scale is to be expected. Values for constant parameters as well es the coefficients for each scale and item of the HPQ28 can be found in Table 4 of the main manuscript. The following examples further illustrate the calculation of normative HPQ28 values in different patient population.

Example 1

Patient sample with a mean age of 65 years, 50% female.

Normative HPQ28 values according to Table 4 of the main manuscript:

PaC: *-1.04 + 0.50 x (0.369) + 65 x (0.065) = 3.4*

NVS: *-0.28 + 0.50 x (0.422) + 65 x (0.018) = 1.2*

LoV: *1.31 + 0.50 x (0.904) + 65 x (0.103) = 8.4*

DaA: *0.49 + 0.50 x (0.761) + 65 x (0.016) = 1.9*

GiS: *0.364 + 0.50 x (0.246) + 65 x (-0.001) = 0.4*

NT: *-0.160 + 0.50 x (0.069) + 65 x (0.008) = 0.4*

TM: *-0.226 + 0.50 x (0.079) + 65 x (0.009) = 0.4*

RH: *-0.187 + 0.50 x (0.075) + 65 x (0.008) = 0.4*

PHQ-2: *0.089 + 0.50 x (0.227) + 65 x (0.013) = 1.0*

Example 2

45-year-old female patient with hypoparathyroidism.

Normative HPQ28 values according to Table 4 of the main manuscript:

PaC: *-1.04 + 1 x (0.369) + 45 x (0.065) = 2.3*

NVS: *-0.28 + 1 x (0.422) + 45 x (0.018) = 1.0*

LoV: *1.31 + 1 x (0.904) + 45 x (0.103) = 6.8*

DaA: *0.49 + 1 x (0.761) + 45 x (0.016) = 2.0*

GiS: *0.364 + 1 x (0.246) + 45 x (-0.001) = 0.6*

NT: *-0.160 + 1 x (0.069) + 45 x (0.008) = 0.3*

TM: *-0.226 + 1 x (0.079) + 45 x (0.009) = 0.3*

RH: *-0.187 + 1 x (0.075) + 45 x (0.008) = 0.2*

PHQ-2: *0.089 + 1 x (0.227) + 45 x (0.013) = 0.9*

**Supplemental Tables**

**Supplemental Table 1** Percent ranks of HPQ28 scales and single items by gender and age

|  | **Male** | | | | **Female** | | | |
| --- | --- | --- | --- | --- | --- | --- | --- | --- |
| Age (years) | <40 | 40-60 | >60 | All | <40 | 40-60 | >60 | All |
| N | 397 | 471 | 362 | 1230 | 412 | 511 | 353 | 1276 |
| **PaC** |  |  |  |  |  |  |  |  |
| Mean±  SD | 0.9±1.7 | 2.1±2.5 | 3.6±2.9 | 2.2±2.6 | 1.3±2.3 | 2.4±2.7 | 4.1±3.0 | 2.5±2.7 |
| 25% | 0 | 0 | 1 | 0 | 0 | 0 | 2 | 0 |
| 50% | 0 | 1 | 3 | 1 | 0 | 2 | 4 | 2 |
| 75% | 1 | 3 | 5 | 4 | 2 | 4 | 6 | 4 |
| 90% | 4 | 5 | 8 | 6 | 4 | 6 | 8 | 7 |
| 95% | 4 | 7 | 9.2 | 8 | 6.8 | 8 | 9.9 | 8 |
| **NVS** |  |  |  |  |  |  |  |  |
| Mean±  SD | 0.3±0.9 | 0.6±1.4 | 0.9±1.7 | 0.6±1.4 | 0.7±1.6 | 1.0±1.6 | 1.4±2.0 | 1.0±1.8 |
| 25% | 0 | 0 | 0 | 0 | 0 | 0 | 0 | 0 |
| 50% | 0 | 0 | 0 | 0 | 0 | 0 | 1 | 0 |
| 75% | 0 | 1 | 1 | 1 | 1 | 1 | 2 | 1 |
| 90% | 1 | 2 | 3 | 2 | 2 | 3 | 4.8 | 3 |
| 95% | 2 | 4 | 5 | 4 | 4.8 | 5 | 6 | 5 |
| **LoV** |  |  |  |  |  |  |  |  |
| Mean±  SD | 4.3±3.6 | 6.3±4.4 | 9.0±4.1 | 6.5±4.5 | 5.6±4.0 | 6.9±4.3 | 9.8±4.3 | 7.3±4.5 |
| 25% | 2 | 3 | 6 | 3 | 3 | 4 | 7 | 4 |
| 50% | 4 | 6 | 8 | 6 | 5 | 6 | 10 | 7 |
| 75% | 6.75 | 9 | 12 | 9 | 8 | 9 | 13 | 10 |
| 90% | 9 | 13 | 15 | 13 | 12 | 14 | 16 | 14 |
| 95% | 11 | 15 | 16 | 15 | 13 | 16 | 16 | 16 |
| **DaA** |  |  |  |  |  |  |  |  |
| Mean±  SD | 1.0±2.2 | 1.3±2.1 | 1.6±2.3 | 1.3±2.2 | 1.7±2.8 | 1.9±2.8 | 2.4±3.0 | 2.0±2.7 |
| 25% | 0 | 0 | 0 | 0 | 0 | 0 | 0 | 0 |
| 50% | 0 | 0 | 1 | 0 | 0 | 1 | 1 | 1 |
| 75% | 1 | 2 | 2 | 2 | 3 | 3 | 4 | 3 |
| 90% | 3 | 4 | 5 | 4 | 6 | 6 | 7 | 6 |
| 95% | 5 | 6 | 6.2 | 6 | 8 | 8 | 9 | 9 |
| **GiS** |  |  |  |  |  |  |  |  |
| Mean±  SD | 0.3±0.7 | 0.3±0.8 | 0.3±0.7 | 0.3±0.8 | 0.6±1.1 | 0.5±0.9 | 0.5±1.0 | 0.5±1.0 |
| 25% | 0 | 0 | 0 | 0 | 0 | 0 | 0 | 0 |
| 50% | 0 | 0 | 0 | 0 | 0 | 0 | 0 | 0 |
| 75% | 0 | 0 | 0 | 0 | 1 | 1 | 1 | 1 |
| 90% | 1 | 1.5 | 1 | 1 | 2 | 2 | 2 | 2 |
| 95% | 2 | 2 | 2 | 2 | 3 | 2 | 3 | 3 |
| **NT** |  |  |  |  |  |  |  |  |
| Mean±  SD | 0.1±0.3 | 0.2±0.6 | 0.4±0.6 | 0.2±0.5 | 0.2±0.5 | 0.3±0.6 | 0.5±0.7 | 0.3±0.6 |
| 25% | 0 | 0 | 0 | 0 | 0 | 0 | 0 | 0 |
| 50% | 0 | 0 | 0 | 0 | 0 | 0 | 0 | 0 |
| 75% | 0 | 0 | 1 | 0 | 0 | 0 | 1 | 0 |
| 90% | 0 | 1 | 1 | 1 | 1 | 1 | 1 | 1 |
| 95% | 0 | 1 | 2 | 1 | 1 | 1 | 2 | 2 |
| **TM** |  |  |  |  |  |  |  |  |
| Mean±  SD | 0.1±0.3 | 0.2±0.5 | 0.5±0.7 | 0.2±0.5 | 0.2±0.5 | 0.3±0.5 | 0.5±0.7 | 0.3±0.6 |
| 25% | 0 | 0 | 0 | 0 | 0 | 0 | 0 | 0 |
| 50% | 0 | 0 | 0 | 0 | 0 | 0 | 0 | 0 |
| 75% | 0 | 0 | 1 | 0 | 0 | 0 | 1 | 0 |
| 90% | 0 | 1 | 1 | 1 | 1 | 1 | 1 | 1 |
| 95% | 1 | 1 | 2 | 1 | 1 | 1 | 2 | 2 |
| **RH** |  |  |  |  |  |  |  |  |
| Mean±  SD | 0.1±0.3 | 0.2±0.5 | 0.4±0.7 | 0.2±0.5 | 0.2±0.5 | 0.3±0.5 | 0.5±0.7 | 0.3±0.6 |
| 25% | 0 | 0 | 0 | 0 | 0 | 0 | 0 | 0 |
| 50% | 0 | 0 | 0 | 0 | 0 | 0 | 0 | 0 |
| 75% | 0 | 0 | 1 | 0 | 0 | 0 | 1 | 0 |
| 90% | 0 | 1 | 1 | 1 | 1 | 1 | 1 | 1 |
| 95% | 1 | 1 | 2 | 1 | 1 | 1 | 2 | 1 |
| **PHQ-2** |  |  |  |  |  |  |  |  |
| Mean±  SD | 0.5±0.9 | 0.8±1.2 | 1.1±1.4 | 0.8±1.2 | 0.8±1.3 | 0.9±1.3 | 1.3±1.5 | 1.0±1.4 |
| 25% | 0 | 0 | 0 | 0 | 0 | 0 | 0 | 0 |
| 50% | 0 | 0 | 1 | 0 | 0 | 0 | 1 | 0 |
| 75% | 1 | 1 | 2 | 1 | 1 | 2 | 2 | 2 |
| 90% | 2 | 2 | 3 | 2 | 2 | 2 | 4 | 3 |
| 95% | 2 | 4 | 4 | 3 | 4 | 4 | 4 | 4 |
| **Total** |  |  |  |  |  |  |  |  |
| Mean±  SD | 7.5±8.6 | 12.0±10.6 | 17.7±11.4 | 12.3±11.0 | 11.3±11.4 | 14.5±12.0 | 20.9±13.2 | 15.1±12.7 |
| 25% | 2 | 5 | 9 | 4 | 4 | 6 | 11 | 6 |
| 50% | 5 | 8 | 15 | 9 | 7 | 11 | 18 | 12 |
| 75% | 10 | 17 | 23 | 17 | 15 | 20 | 29 | 22 |
| 90% | 18 | 27 | 34.4 | 27 | 26.6 | 33 | 39.8 | 34 |
| 95% | 22.75 | 33 | 40 | 35 | 36 | 40 | 48 | 41.8 |

DaA = depression and anxiety, GiS = gastrointestinal symptoms, HPQ28 = 28 item Hypoparathyroidism Patient Questionnaire, LoV = loss of vitality, NT = numbness or tingling, NVS = neurovegetative symptoms, PaC = pain and cramps, PHQ-2 = 2 item Patient Health Questionnaire, RH = racing heart, SD = standard deviation, TM = troubled memory
